# Comment on “A simple tool for neuroimaging data sharing”

**DOI:** 10.3389/fninf.2014.00082

**Published:** 2014-10-30

**Authors:** Christian Haselgrove, Jean-Baptiste Poline, David N. Kennedy

**Affiliations:** ^1^Department of Psychiatry, University of Massachusetts Medical SchoolWorcester, MA, USA; ^2^Henry H. Wheeler, Jr. Brain Imaging Center, University of California at BerkeleyBerkeley, CA, USA

**Keywords:** neuroinformatics, neuroimaging, DICOM, data archiving, data sharing, anonymization

In our recent paper “A simple tool for neuroimaging data sharing,” we introduced a system for sharing DICOM data. Addressing anonymization, we mentioned DICOM Supplement 55^1^, the National Cancer Institute deidentification profile, and the default deidentification profile in XNAT's DICOM Browser and noted the disagreement in these various anonymization profiles. While a careful analysis of anonymization (especially as applied to DICOM) was not in the scope of this work, we could also have mentioned further work from the DICOM Standards Committee, specifically Supplement 142^2^ (Clinical Trial De-identification Profiles) and Annex E (Attribute Confidentiality Profiles) of PS3.15^3^ (Security and System Management Profiles), which provide well thought-out and detailed analyses and recommendations for anonymization of DICOM data by dedicated working groups.

Also, in our observation of the current state of DICOM anonymization within the neuroimaging research community, we stated that no consensus could be found. Certainly most solutions in the neuroimaging research community do not follow the DICOM standard, preferring instead to design their own schemes that satisfy different levels of anonymization needed given each specific Institutional Review Board's (IRB) requirements and the nature of the specific data; the result is a lack of consensus in this particular community. This is an unfortunate reality and should not be construed to reflect negatively on the effort and the outcomes of the DICOM working groups, which are consensus solutions from the broader imaging community.

Indeed, there are several tools that support the DICOM standards out of the box, among these dicom-anon^4^ (supporting PS3.15, Annex E), DICOM Anonymizer^5^ (PS3.15, Annex E), the CTP DICOM Anonymizer^6^ (Supplement 142), and gdcmanon^7^ (PS3.15, Annex E and Supplement 142). However, these tools have been developed for radiological or more general biomedical research applications and the authors have not seen them adopted by the neuroimaging community. Tools such as DicomBrowser^8^, part of the XNAT environment more familiar to neuroimagers, tend to focus on flexible anonymization, and configuration files supporting PS3.15, Annex E are available but must be downloaded separately.

By integrating all of these considerations regarding anonymization profiles, we hope that the neuroimaging research community will also begin to converge on standardization of this important aspect of data sharing.

## Conflict of interest statement

The authors declare that the research was conducted in the absence of any commercial or financial relationships that could be construed as a potential conflict of interest.

## Footnotes

^1^Digital Imaging and Communications in Medicine (DICOM). Supplement 55: Attribute Level Confidentiality (including De-identification). Available online at: http://medical.nema.org/Dicom/supps/sup55_03.pdf

^2^Digital Imaging and Communications in Medicine (DICOM). Supplement 142: 10 Clinical Trial De-identification Profiles. Available online at: ftp://medical.nema.org/medical/dicom/Final/sup142_ft.pdf

^3^NEMA PS3/ISO 12052, Digital Imaging and Communications in Medicine (DICOM) Standard, National Electrical Manufacturers Association, Rosslyn, VA, USA. Available online at: http://dicom.nema.org/dicom/2013/output/chtml/part15/PS3.15.html

^4^https://github.com/cbmi/dicom-anon

^5^http://doradiology.com/DICOManonymizer/

^6^http://mircwiki.rsna.org/index.php?title=The_CTP_DICOM_Anonymizer

^7^http://gdcm.sourceforge.net/html/gdcmanon.html

^8^http://nrg.wustl.edu/software/dicom-browser/

